# Solidarity with Animals: Assessing a Relevant Dimension of Social Identification with Animals

**DOI:** 10.1371/journal.pone.0168184

**Published:** 2017-01-03

**Authors:** Catherine E. Amiot, Brock Bastian

**Affiliations:** 1 Department of Psychology, Université du Québec à Montréal, Montréal, Québec, Canada; 2 Melbourne School of Psychological Sciences, The University of Melbourne, Melbourne, Victoria, Australia; University of Missouri Columbia, UNITED STATES

## Abstract

Interactions with animals are pervasive in human life, a fact that is reflected in the burgeoning field of human-animal relations research. The goal of the current research was to examine the psychology of our social connection with other animals, by specifically developing a measure of solidarity with animals. In 8 studies using correlational, experimental, and longitudinal designs, solidarity with animals predicted more positive attitudes and behaviors toward animals, over and above existing scales of identification, and even when this implied a loss of resources and privileges for humans relative to animals. Solidarity with animals also displayed predicted relationships with relevant variables (anthropomorphism, empathy). Pet owners and vegetarians displayed higher levels of solidarity with animals. Correlational and experimental evidence confirmed that human-animal similarity heightens solidarity with animals. Our findings provide a useful measure that can facilitate important insights into the nature of our relationships with animals.

## Introduction

‘‘*If part of the other resides within us*, *if we feel one with the other*, *then improving their life automatically resonates with us*”–Frans de Waal (2009, pp. 116–117)

Due to thousands of years of cohabitation, a strong interdependence exists between humans and other species [1] and this interdependence has significant psychological implications for humans [2]: Animals entertain us, they are represented in various forms of art, and they have been used as emblems and symbols of human attributes (e.g. [3–5]). Animals feature prominently in the socialization of children [6]. Two out of three Americans live with animals, spending more than $55 billion annually on their welfare (e.g., food, veterinary care, animal purchases, pet services) [7]. More broadly, there is a growing social movement in favor of recognizing animals’ places in human lives and in protecting their interests and habitats [8–9]. Yet, our relations with animals can also be characterized by domination and intergroup conflict [10]. We currently eat a particularly high number of animals (approximately 9 billion each year in the US) [11] and use animals for clothing, for testing a range of human products, and for gaining basic insights into human biology and behavior. Human-animal relations are an important domain of human activity [12]; it is an emerging field of research in psychology, and a diversity of scientific disciplines pay increased attention to this theme, including sociology, economics, geography, history, literature, and philosophy (for an overview of research conducted across these disciplines, please see [13]; and also [14]. Whereas our interactions with animals are known to have physiological and evolutionary bases, as proposed in the biophilia hypothesis [15–17], little is known about the specific *psychological* links that exist between humans and animals, how we feel connected to them, and how we subjectively conceive of our shared evolutionary roots [18].

To approach this question, we investigate the extent to which humans can feel solidarity with other animals–i.e., defined as the sense of belonging, psychological attachment, and closeness felt toward other animals [19]–using an intergroup relations perspective [20–21]. More specifically, we aimed to: (a) adapt and develop a new measure to assess solidarity with animals–as a particularly relevant dimension of identification with animals, (b) understand the nature of, and the processes that trigger, solidarity with animals, and (c) verify how this sense of solidarity has consequences not only for how we perceive and treat animals, but also for resource decision-making and for animal-rights activism. By doing so, our research contributes to the field of human-animal relations, providing new insight into the nature of our psychological connection with other animals.

### Prior Research Relevant to the Notion of Solidarity with Animals

Prior research has hinted at the possibility for a sense of connection and belongingness that extends beyond human groups. Work investigating the notion of identification with nature–defined as the connection that people have to some part of the nonhuman natural environment [22]–has made some progress in this respect. Also in the environmental literature, a general process of self-transcendence and altruism is proposed to take place; this process allows us (humans) to extend beyond our conspecifics and to develop concerns, attitudes, and values that favor the broader environment [23–24] as well as non-human species and the biosphere [25].

Yet, a specific focus on how humans feel connected to other animals is needed given the recent surge of interest in human-animals relations (see [13]). Within this literature, Serpell [26] proposed the notion of ‘‘the animal within”, highlighting the part of the human self-concept that ties us to other animals. This connection to animals emerges in early childhood, with children showing a tendency to categorize humans and non-human animals as part of the same category as early as 14-months of age [27] (see also [28]). Throughout the lifespan attachment to animals, and pets in particular, is evident [29–30] and has been shown to have a range of implications, including for human mental health [31]. In the current paper we extend on this work by examining human-animal relations from an *intergroup* perspective, focusing on the dynamics that operate between animals and humans as members of broad social categories [21], and more specifically on the solidarity that humans may feel toward other animals.

### An Intergroup Approach to Human-Animal Relations

Employing an intergroup perspective allows us to systematically capture the ‘‘us” vs. ‘‘them” dynamics that also operate in our relationship with animals [10, 21, 32–33]. In the intergroup approach, social identification is defined as ‘‘that part of the individual’s self-concept which derives from his or her knowledge of membership to a social group (or groups) together with the value and the emotional significance attached to it” [34] (p. 255); concretely it represents the psychological link that ties us to the other individuals within a group [35]. Historically, theories of intergroup relations–such as the social identity approach [21]–were developed to bring group and intergroup phenomena to the fore in mainstream psychology, to understand how being placed in a particular social category can then impact on individual group members’ behaviors and points of view, and to account for phenomena such as collective movements and actions (see [36] for an historical overview). Whereas prior social identity research has traditionally focused on how we identify with groups of humans (cf. [22]), herein we seek to extend this work to develop new insights into human-animal relations. Specifically, we raise the following questions: Whereas biologically, humans are considered as part of the animal kingdom [18], to what extent do humans subjectively experience solidarity with animals as a social group? What are the predictors that underpin this sense of connectedness and what are its consequences for both humans and animals?

### Solidarity as a Relevant Dimension of Social Identification with Animals

Based on research that distinguishes between the different dimensions of social identification and their specific outcomes [19, 37–38], in the current research we focus on one particular dimension of social identity, namely the solidarity dimension [19] (see also [39–40]). Based on prior work conducted on social identification, solidarity involves one’s psychological bond with, and commitment to, fellow in-group members [19, 41]. This particular dimension of social identification captures its relational side and the concrete roles that we occupy within a group [42]. It involves investment of the self in coordinated activity with those to whom one feels committed [19]. In this sense, solidarity should be associated with approaching the ingroup and with an increased participation in the group (e.g. [43]), rather than avoidance of the ingroup and its obligations (e.g. [44]).

In the realm of human groups, the solidarity dimension of social identification has been found to predict greater collective esteem and more responsiveness to threats that target the ingroup [19]. Conceptually, this specific dimension of social identity was considered particularly relevant to assess in the context of human-animal relations as it is likely to yield to a strong commitment and positive inclination toward animals, including pets with whom we have concrete, everyday relationships [31], but also the animals who are used for human purposes [45].

In the current paper we hence examine whether solidarity with animals leads to more positive attitudes–such as lower speciesism, or prejudice against animals [9]–and more prosocial behaviors toward them. In line with the opening quote, to the extent that we consider the ‘‘other” (i.e., nonhuman animals) as close to the self, we may also be more likely to take their perspective and interests to heart, to adopt more positive attitudes and behaviors toward them, and even to engage in collective actions on their behalf [46]–an issue which is currently gaining increased momentum and social importance [8–9]. Furthermore, solidarity with animals may even lead people to become less prejudiced toward other human outgroups. Indeed, prior research suggests that when animals are framed as more similar to humans, people not only report lower prejudice toward animals, but also toward maligned human outgroups [47–48]. These findings suggest that thinking of how animals are similar to humans possibly triggers the recategorization of both non-human animals and humans into a very inclusive superordinate social group, which cognitively also includes devalued human outgroups. Studies conducted at the interpersonal level of analysis have also revealed that being in the presence of an animal can promote more positive human-to-human contacts [49–50]; see also [13] for a review. However, at the intergroup level, whether feeling solidarity with animals–as a dimension of social identification–is associated with lower bias even toward human outgroups has yet to be captured directly.

### The Present Research

In order to examine solidarity with animals, we sought to learn more about the nature of this construct, establish the psychometric properties of a newly developed solidarity with animals measure, and test its antecedents and correlates. Eight studies were conducted to achieve these goals. Studies 1 and 2 test the convergent, divergent, and predictive validity of the solidarity with animals measure over and above relevant established measures that also assess highly inclusive social identities (i.e., human identity; identification with nature). Study 3 examines whether solidarity with animals is associated with lower prejudice, even toward human outgroups. Studies 4a and 4b examine criterion validity, focusing on pet compared to non-pet owners and meat-eaters compared to non-meat eaters. Study 5 employs an experimental design to test the role of human-animal similarity (vs. difference) as a relevant antecedent to solidarity with animals. Study 6 tests the nomological validity of the solidarity with animals measure, further establishing similarity as an antecedent to solidarity with animals and reduced prejudice against animals as a consequence. Finally, Study 7 provides further evidence of predictive validity for the measure by longitudinally testing the relationship between solidarity with animals and reduced prejudice against animals and resource decision making 5 months later. Table 1 provides a brief description of these studies.

**Table 1:** Main Goals, Variables and Findings of the Studies.

Study	Goal	Main Variables	Findings
Study 1	Assess the predictive power of the Solidarity with Animals Scale relative to other identification scales	Solidarity with animals Solidarity with humans Identification with nature Moral concerns toward animals Speciesism	Solidarity with animals was correlated positively with identification with nature (but not with solidarity with humans). Solidarity with animals (but not solidarity with humans or identification with nature) negatively predicted speciesism. Solidarity with animals and identification with nature (but not solidarity with humans) positively predicted moral concerns for animals
Study 2	Test the convergent and divergent validity of the Solidarity with Animals Scale	Solidarity with animals Dispositional empathy Anthropomorphism Openness to experience Pet attachment	Solidarity with animals was correlated positively with: dispositional empathy, openness to experience, anthropomorphizing animals and nature (but not technology), anxious attachment to pet. Solidarity with animals was correlated negatively with: avoidant attachment to pet
Study 3	Test the associations between solidarity with animals and human prejudices	Solidarity with animals Sexism Racism Ageism Gender Political beliefs Socio-economic status	Solidarity with animals was associated with lower: ageism, right wing authoritarianism, racism, sexism (only among women), more likelihood of holding left-leaning political beliefs, and being a woman. Solidarity with animals was not associated with: age, religion, socio-economic status
Studies 4a and 4b	Test the ability of the scale to differentiate between pet vs. non pet owners (4a) and vegetarians vs. non vegetarians (4b)	Being a pet owner vs. not Number of pets Pet species Being vegetarian vs. meat eater Meat eating frequency	Pet owners and vegetarians reported higher solidarity with animals than non pet owners and non-vegetarians Solidarity with animals correlated positively with the number of pets and negatively with meat eating frequency. Cat and dog owners did not differ in their solidarity with animals.
Study 5	Test if experimentally activating human-animal similarity (vs. difference) increases (vs. decreases) solidarity with animals	Three conditions:- Animals as similar to humans Animals as different from humans, Control Assessment of solidarity with animals	Participants in the similarity condition reported higher solidarity with animals than those in the difference and in the control conditions.
Study 6	Establish nomological validity with relevant constructs	Solidarity with animals Perception of human-animal similarity Attitudes toward animals (i.e., moral concerns for animals; speciesism; justification for the use of animals)	Solidarity with animals mediated the links between perceived human-animal similarity (as the cognitive antecedent) and the attitudes toward animals variables (as the outcomes)
Study 7	Test the predictive validity of the Solidarity with Animals Scale using a two-wave longitudinal design (5-month time gap)	Solidarity with animals Attitudes toward animals (i.e., moral concerns for animals; justification for the use of animals; speciesism) Bias in favor of humans over animals in general and over pets (i.e., feeling thermometers; positive adjectives; money donation; moral dilemmas). Animal rights activism Collective action intentions	Time 1 solidarity with animals was associated with lower Time 2: speciesism, justification for the use of animals, bias in favor of humans over animals and over pets, tendency to favor humans over dogs. Time 1 solidarity with animals was associated with higher Time 2: moral concern toward animals, intentions to engage in collective actions on behalf of animals, animal rights activism, and donations to animal charities (relative to human charities).

## Predictive Validity: Study 1

Study 1 examines the extent to which the notion of solidarity with animals is endorsed and uniquely predicts attitudes and behaviors toward animals (i.e., lower speciesism and higher moral concerns toward animals), relative to other inclusive superordinate identities including identification with nature and solidarity with humans. Animals being part of nature [22], we expected the notions of solidarity with animals and identification with nature to correlate positively.

### Method

#### Participants

Participants were 148 students from the Université du Québec à Montréal (UQAM; 114 female; *M*^*age*^ = 23.12, *SD* = 4.45). Participants provided their written informed consent (on a paper consent form) to participate in this study. The UQAM ethics committee approved this study.

#### Measures

*Solidarity with animals* was measured using five items. Three items were directly adapted from Leach et al.’s [19] social identification subscale of solidarity: “I feel a strong bond toward other animals”; “I feel solidarity toward animals”; and ‘‘I feel committed toward animals”. To maximize scale reliability and in line with guidelines recommending the use of 5 items to assess a construct [51], two additional items were constructed based on the definition of solidarity [52–53]: ‘‘I feel close to other animals”; ‘‘I feel a strong connection to other animals”. These five items were measured on a 1(*strongly disagree*) to 7(*strongly agree*) likert-type scale (α = .94). *Solidarity with humans* was assessed using the same 5 items (e.g., α = .91; “I feel a strong bond toward other humans”).

We employed four items from the Collective Self-Esteem Scale [54] adapted to assess *identification with nature* [22]. Items were measured on a 1(*strongly disagree*) to 7(*strongly agree*) likert-type scale (“I think of myself as part of nature, not separate from it”; “I feel that I have a lot in common with other species”; ‘‘Being part of the ecosystem is an important part of who I am”; ‘‘In general, being part of the natural world is an important part of my self-image”; α = .87).

In terms of animal-related outcomes, *moral concern toward animals* was measured by asking participants to select from a list of 25 animals which ones they feel morally obligated to show concern for [55]. A score out of 25 was calculated for the number of animals participants felt morally obligated to show concern for. *Speciesism* was measured using twenty items, including ten items from the Animals Rights Scale [56], and ten items from the Animal Attitudes Scale [57]. Items were aggregated to create a total 20-item ‘‘speciesism” scale (α = .87). Fourteen items were positively worded (e.g., “I think it is perfectly acceptable for cattle, chickens and pigs to be raised for human consumption”) and six items were reversed scored (“Animal research cannot be justified and should be stopped”). The scores were averaged with higher scores indicating greater speciesism. Items were measured on a 1(*do not agree at all*) to 7(*agree completely*) likert-type scale. All measures for this study as well as those conducted among UQAM students were completed in French.

### Results and Discussion

#### The solidarity with animals construct

The descriptive statistics for each item of the solidarity with animals scale can be seen in Table 2. Principle component analyses on the five solidarity with animals items yielded a robust single factor solution, eigenvalue = 4.05 (accounting for 80.90% of variance), with item loadings on this factor ranging from .85 to .94 (see Table 2). Inspection of the scree plot also indicated a single-factor solution. To ensure that our solidarity with animals measure was statistically independent from other broader identification concepts, we conducted a principal component analysis on the five solidarity with animals items, the five solidarity with humans items, and the four identification with nature items. A three-factor solution emerged, eigenvalue = 10.73 (accounting for 76.73% of variance), with all five items of the solidarity with animals scale loading on the first factor (ranging from .78 to .90), while the five items from the solidarity with humans measure loaded on the second factor (from .78 to .92), and the four items from the identification with nature measure loaded on the third factor (from .71 to .87).

**Table 2:** Means, Standard Deviations, and Factor Loadings for the Items of the Solidarity With Animals Measure (Study 1).

	Descriptive Statistics		*Factor Loading*
Items	*M*	*SD*	
Item 1: I feel a strong bond toward other animals	4.97	1.54	.90
Item 2: I feel solidarity toward animals	4.84	1.57	.88
Item 3: I feel close to other animals	4.57	1.83	.93
Item 4: I feel a strong connection to other animals	4.17	1.89	.94
Item 5: I feel committed toward animals	4.35	1.84	.85

*M* = Mean; *SD* = Standard Deviation.

Convergent and divergent validity were assessed by examining the associations between the solidarity with animals scale and the measures of identification with nature and solidarity with humans. Solidarity with animals correlated positively with the identification with nature measure (*r* = .59, *p* < .001; 95% CI [.46, .72]). However, a non-significant association emerged between solidarity with animals and solidarity with humans (*r* = -.02, *p*>.05; 95% CI [-.17, .13]). This null association could be explained by the fact that solidarity with animals and with humans are distinct and non-overlapping constructs. For instance, the decision to be invested in one group is not tied to one’s solidarity toward the other. This potential independence of the subgroup (human) and superordinate (animal) identities has also been noted by prominent intergroup relations theorists [20]. We return to a discussion of the interplay between subgroup and superordinate identities in the General Discussion section.

A repeated-measures ANOVA revealed that respondents on average reported different scores on the measures of solidarity with animals, solidarity with humans, and identification with nature (*F*(2,144) = 12.41, *p* < .001, ƞ_p_^2^ = .15). Planned contrasts showed that participants reported similar scores on the solidarity with animals (*M* = 4.57, *SD* = 1.57) and identification with nature measures (*M* = 4.75, *SD* = 1.37; *F*(1,145) = 2.59, *p* = .110, ƞ_p_^2^ = .02; 95% CI [-.09, .44]), but they reported lower scores on the solidarity with animals measure than on the solidarity with humans measure (*M* = 5.35, *SD* = 1.05; *F*(1,145) = 24.13, *p* < .001, ƞ_p_^2^ = .14; 95% CI [-.1.16, -.39]). The mean score for solidarity with animals was also significantly higher than the scale midpoint of 4 (i.e., *moderately agree*; *t*(1,147) = 4.54, *p* < .001; 95% CI [.33, .83]), suggesting, on average, at least moderate endorsement of the solidarity with animal construct.

#### The predictive validity of solidarity with animals

We compared the solidarity with animals scale to the other superordinate identification scales (i.e., identification with nature, solidarity with humans), as predictors of the animal-related outcome variables (speciesism, moral concern for animals). As can be seen in Table 3, when predicting speciesism, solidarity with animals, as well as solidarity with humans and identification with nature accounted for a significant amount of the variance in speciesism (*R*^2^ = .38, *F*(3,142) = 28.54, *p* < .001). However, only solidarity with animals (*β* = -.60, *p* < .001; 95% CI [-.70, -.40]) significantly negatively predicted speciesism. Solidarity with animals, as well as solidarity with humans and identification with nature also accounted for a significant amount of the variance in moral concerns for animals (*R*^2^ = .40, *F* (3,142) = 31.22, *p* < .001). Solidarity with animals (*β* = .41, *p* < .001; 95% CI [.24, .55]) and identification with nature (*β* = .28, *p* < .01; 95% CI [.12, .44]) positively predicted moral concerns for animals. This last association is consistent with the fact that animals are generally considered a part of nature.

**Table 3:** Multiple regressions predicting animal-related outcomes from the solidarity with animals and the superordinate identity measures (Study 1).

	Regression Coefficient (B)	
Variable	Speciesism	Moral Concern toward Animals
Solidarity with animals	-.60***	.41***
Solidarity with Humans	-.06	-.12
Identification with Nature	-.02	.28**

**p < .01.

***p < .001.

The findings confirm that on average participants endorse at least moderately the solidarity with animals construct. The solidarity with animals construct also correlated positively with identification with nature, as another inclusive social identity. Yet when predicting animal-related outcomes (speciesism, moral concerns for animals), the solidarity with animals measure was the more constant predictor, providing support for the discriminant validity of the solidarity with animals measure and its capacity to predict conceptually aligned outcomes.

## Convergent and Divergent Validity: Study 2

Study 2 was designed to test the associations between solidarity with animals and the constructs of dispositional empathy, anthropomorphism, attachment to a pet, and openness to experience. These constructs are particularly relevant to human-animal relations. Indeed, empathy–which involves cognitively taking another person’s perspective and being emotionally sensitive to their suffering [58]–has been linked to positive attitudes toward animals [59] as well as greater concern regarding animal cruelty [60]. We extend these findings by testing if dispositional empathy (and its different components) is associated positively with the notion of solidarity with animals. Anthropomorphism [61] is also relevant to human-animal relations given that it involves cognitively projecting human characteristics onto nonhuman animals. Specifically, we expect that solidarity with animals will relate to a tendency to anthropomorphize animals and elements of the natural environment, but not machines. This expected pattern of correlations would provide evidence for the discriminant validity of the solidarity with animals measure. We also expected that solidarity with animals would relate positively to openness to experience [62]. Indeed, in the human-animal relations literature, personality variables related to creativity and unconventionality (e.g., intuition, imaginativeness)–which are also associated with openness to experience–have been associated with more positive attitudes toward animals [63–64]. Broad superordinate identities–such as the one captured by the measure of solidarity with animals–are also typically associated with more cognitive flexibility and a broader thinking style [65].

Finally, we also explored how solidarity with animals is (negatively) associated with avoidant and anxious attachment to pets using a recently constructed attachment to pets scale [31]. Research has revealed that companion animals can serve as attachment figures for their owners [66]. We expected that solidarity with animals would be related to both lower avoidant and lower anxious attachment as it should promote direct engagement with animals as a group (see [44]), including pets.

### Method

#### Measures

The same five items as in Study 1 were used to assess solidarity with animals (α = .93). To assess *dispositional empathy*, the Interpersonal Reactivity Index [58] was employed. Its four subscales (7-items each) of perspective taking, fantasy, empathic concerns, and personal distress showed adequate reliability (αs = .78, .77, .78, & .83, respectively; overall reliability = .81). To assess *anthropomorphism*, we used the Individual Differences in Anthropomorphism Questionnaire [67]. The three subscales assessing anthropomorphism of technology, nature, and animals showed adequate reliability (5-items each; αs = .63, .90, and .85, respectively; overall reliability = .84). *Openness to experience* was assessed by employing the Big Five Inventory Personality Test’s openness to experience subscale (10 items: α = .75) [68]. Finally, *pet attachment* to a current or past pet was assessed using the Pet Attachment Questionnaire [31]: Both attachment avoidance and anxiety subscales presented adequate reliabilities (13 items each: αs = .80 and .84, respectively; overall reliability = .81).

#### Participants and procedures

Participants were recruited online through the websites and Facebook pages of different Canadian, American, and Australian universities (e.g., University of Queensland, University of Florida, University of Toronto), animal-related organisations (e.g., SPCA, Canadian Kennel Club, Vegetarian Page, Hunting and Fishing New Zealand, Pet Owners Association), and general psychology interest groups (e.g., Social Psychology Network, Psychology Today). The sample included 365 participants (286 women, *M*^age^ = 31.27, *SD* = 13.54). Participants provided their informed consent by clicking on a box in the online consent form confirming that they had read the consent form and agreed to take part in the study. The UQAM ethics committee approved this study.

### Results and Discussion

As shown in Table 4, solidarity with animals correlated positively with dispositional empathy (and in particular its empathic concern subscale), anthropomorphism, and openness to experience. Providing convergent and divergent validity for the solidarity with animals construct, and as expected, solidarity with animals was associated positively with anthropomorphizing animals and nature, but not with anthropomorphizing technology. These correlations were in the small to moderate range, confirming that solidarity with animals is not redundant with these other measures. As expected, greater solidarity with animals was associated with lower avoidance attachment toward pets [44]; however, solidarity with animals was associated positively with attachment anxiety. The relationships between solidarity with animals and the pet attachment styles suggest that people high in solidarity may be more likely to generally hold close to animals, rather than to distance themselves from animals, even if this implies higher feelings of anxiety or dependency toward their pet. This positive link between the solidarity dimension of social identification and an enhanced sensitivity to anxiety/group threat was also uncovered in research focusing on human-human intergroup relations [19]. Future research could seek to uncover the specific processes that underpin this positive link, such as an increased need to affiliate with members of this group, and the fear associated with losing one of its valued members (such as one’s pet [69].

**Table 4:** Correlations between solidarity with animals and empathy, anthropomorphism, openness to experience, and attachment to pets (Study 2).

Heading	Heading	Solidarity with Animals
**Dispositional Empathy–Overall Score**		.20***
	Empathy-Perspective Taking	.10
	Empathy-Fantasy	.09
	Empathy-Empathetic Concerns	.30***
	Empathy-Personal Distress	.02
**Anthropomorphism–Overall Score**		.22***
	Anthropomorphism-Technology	-.05
	Anthropomorphism-Nature	.12*
	Anthropomorphism-Animals	.32***
**Openness to Experience**		.15**
**Avoidant Attachment to Pet**		-.47***
**Anxious Attachment to Pet**		.19***

*p < .05.

**p < .01.

***p < .001.

## Solidarity with Animals and Human Prejudice: Study 3

Study 3 tested the associations between solidarity with animals and different forms of prejudice (i.e., sexism, racism, ageism) as well as with ideological beliefs theorized to underpin prejudice and ingroup bias: namely right wing authoritarianism (RWA) [70] and social dominance orientation (SDO) [71]. Based on research showing that greater human-animal similarities promote heightened concerns even for human outgroups [47–48], we expected that solidarity with animals–because it taps into a broad and inclusive sense of social connection–should be associated with lower prejudice towards other humans. Providing evidence for this association would allow us to go beyond prior work by showing how social identification processes per se–in this case, the dimension of solidarity with animals–underpin lower prejudice toward humans. Given the cognitive flexibility and openness that characterizes solidarity with animals, this construct should also be associated negatively with SDO and RWA.

### Method

#### Measures

The same five items were used to assess *solidarity with animals* (α = .95). *Sexism* was assessed using Glick and Fiske’s [72] Ambivalent Sexism Inventory (αs = .94 and .81 for the 11-item each hostile and benevolent sexism subscales, respectively; 1 = *strongly disagree*; 6 = *strongly agree*), and Swim, Aikin, Hall, and Hunter’s [73] Modern Sexism Scale (8-items, α = .74; 1 = *strongly agree;*5 = *strongly disagree*–scores were recoded to indicate higher sexism). To assess *racism* and *ageism*, the Symbolic Racism Scale [74] (α = .83, 8-items; response scales used for each item are in line with original measure) and the Succession, Identity, and Consumption Scale of Prescriptive Ageism measure [75] (20 items; α = .93; 1 = *disagree strongly*; 6 = *agree strongly*) were also used. *SDO* and *RWA* were assessed using the measures developed by Pratto et al. [71] (α = .97, 16-items; 1 = *very positive*; 7 = *very negative–* scores were recoded to indicate higher SDO) and Altemeyer [70] (α = .97, 22 items; 1 = *very strongly agree*; 9 = *very strongly disagree*—scores were also recoded), respectively.

#### Participants and procedures

The participants were recruited from Mechanical Turk [76]. Participants provided their informed consent by clicking on a box in the online consent form confirming that they had read the consent form and agreed to take part in the study. The UQAM ethics committee approved this study. The main analyses focused on the 172 non-Black participants (74 women, *M*^age^ = 37, *SD* = 13.90), given that racism measures have been found to operate best among White participants [74]. Whereas 38% of participants followed a religion, 62% did not. On average, religion was moderately important for participants in their daily lives (*M* = 4.91; *SD* = 1.55, on a 7-point scale ranging from 1(*not at all important*) to 7(*extremely important*). Participants were located on the middle left with respect to their political beliefs on the economy (social welfare, government spending, tax; *M* = 3.31, *SD* = 1.50) and their political beliefs on social issues (immigration, homosexual marriage, abortion; *M* = 2.63, *SD* = 1.55, both assessed on a 7-point scale ranging from 1 = *left/liberal* to 7 = *right/conservative*. Mean rating on a 10-point socioeconomic status ladder was 4.88 (*SD* = 1.79).

### Results and Discussion

As expected, solidarity with animals correlated negatively with different forms of prejudice, namely lower symbolic racism (*r* = -.15, *p* = .057; 95% CI [-.39, .02]) and ageism (*r* = -.27, *p* < .001; 95% CI [-.42, -.13]), as well as lower endorsement of RWA (*r* = -.26, *p* = .001; 95% CI [-.41, -.11]) and SDO (*r* = -.16, *p* = .043; 95% CI [.01, .31]). Demographically and politically, solidarity with animals correlated with a tendency to endorse left-leaning (or liberal) political orientations on social (*r* = -.25, *p* = .001; 95% CI [-.38, -.10]) and economic (*r* = -.17, *p* = .023; 95% CI [-.31, -.02]) issues, with being female (*r* = -.19, *p* = .013; 95% CI [-.33, -.04]), but not with age (*r* = .09, *p* = .254; 95% CI [-.19, .11]), socioeconomic status (*r* = -.04, *p* = .598; 95% CI [-.19, .11]), adhering to a religion or not (*r* = .04, *p* = .614; 95% CI [-.11, .18]), and the importance of this religion (*r* = -.02, *p* = .881; 95% CI [-.27, .23]). While solidarity with animals did not correlate significantly with the sexism measures among the overall sample (*r*s ranged from -.13 to -.10, all *p*s>.05; 95% CIs ranged from -.27 to .07 and all pairs spanned zero), when conducting the correlational analyses separately for male and female participants, clear negative correlations emerged between solidarity with animals and most sexism measures among female participants (benevolent sexism: *r* = -.33, *p*-.004; 95% CI [-.56, -.11], hostile sexism: *r* = -.19, *p* = .112; 95% CI [-.49, .05], modern sexism: *r* = -.36, *p* = .002; 95% CI [-.70, -.17]) but not among males (benevolent sexism: *r* = .16, *p* = .108; 95% CI [-.03, .34], hostile sexism: *r* = .08, *p* = .440; 95% CI [-.12, .28], modern sexism: *r* = .10, *p* = .340; 95% CI [-.10, .28]).

The findings confirmed that solidarity with animals correlates negatively with certain forms of prejudice, positively with left-leaning political beliefs and with being female. However, solidarity with animals was not associated with age, socioeconomic status, or religion. In line with prior work conducted on human-animal relations [77], women reported higher solidarity with animals. Research on environmental concern also finds that women consistently express slightly greater environmental concern than men [78] and tend to perceive that the quality of the environment is more likely to have consequences on both human well-being and on the broader biosphere [25]. Again this gender difference may be underpinned by differences in general altruistic/transcendence values which appear to apply to both environmental concerns and to concerns for animals.

While we found our predicted association between solidarity with animals and reduced sexism, this was only evident for female but not male participants. This particular finding for women could be due to the facts that sexism is generally less strongly endorsed by women than men [79], and that solidarity with animals is especially associated with liberal (as opposed to conservative) political views in women (for whom political beliefs and solidarity with animals were correlated at *r* = -.25, *p* = .032; 95% CI [-.43, -.02], and *r* = -.38, *p* = .001; 95% CI [-.61, -.16]) relative to men (*r* = -.07, *p* = .520; 95% CI [-.27, .14], and *r* = -.10, *p* = .331; 95% CI [-.28, .10]).

## Criterion Validity: Study 4a

Where might we find groups of individuals who are particularly high in terms of solidarity with animals? An obvious place to look is among pet owners. By definition, pet owners develop a non utilitarian relationship with their animal and endorse specific roles with regards to this animal [80]. Pet owners can display high commitment to their animal, adjusting their lifestyle to suit their animals’ needs (exercise, feeding times), and spending considerable resources (time, money, energy) on their animal [81]. Most pet owners also consider their pet as an integral member of the family [82–83]. This connection and commitment toward a particular subgroup of animals (i.e., pets) could then flow on to predict higher solidarity with animals in general. This is because pets may play the role of ‘‘ambassadors” of other animals and serve as a springboard to increasing concerns toward animals at large [26]. Empirical evidence confirms that concerns for pets as a particular type of animal can generalize to cover concerns for broader species and types of animals, such as lions, pigs, chickens, and snakes [84], and animals used in the fur and leather industry and zoo animals [85]. In Study 4a, we expand these findings to directly test if contact with pets–in the form of pet ownership–is associated with increased solidarity with all animals.

### Method

#### Participants

We compared pet and non-pet owners in a convenience sample for which we naturally observed comparable numbers of pet (*n* = 64) and non-pet owners (*n* = 50; two did not report pet ownership information). This total sample included 116 participants (69 were females; *M*^age^ = 28.74, *SD* = 11.58) recruited from around the Université du Québec à Montréal and within its immediate vicinity (i.e., waiting room of the university registrar office, shopping mall). Participants completed the questionnaire in a quiet environment with the research assistant nearby. Among pet owners, the majority of participants (66%) currently had 1 pet (*M* = 1.52 pet; *SD* = 1.05). Participants provided their written informed consent (on a paper consent form) to participate in this study. The UQAM ethics committee approved this study. A manipulation that portrayed animals in a more vs. less human-like manner was included in an independent phase prior to measuring the main variables but it did not impact on these variables.

### Results and Discussion

As expected, participants who have pets reported significantly higher solidarity with animals (*M* = 4.52; *SD* = 1.41) than those who do not have pets (*M* = 3.18; *SD* = 1.20; *F*(1,112) = 28.44, *p <* .001, ƞ_p_^2^ = .203; 95% CI [-1.83, -.84]). In addition, solidarity with animals correlated significantly with the number of pets participants currently had (*r* = .33, *p* < .001; 95% CI [.15, .51]). Given prior evidence revealing differences between people who prefer dogs vs. cats [86], we compared people who owned cats (*n* = 27) vs. dogs (*n* = 26) in terms of their levels of solidarity with animals. For participants who had more than one pet, the first pet they listed was employed to categorize them as a dog vs. cat owner. Cat and dog owners did not differ on the solidarity with animals measure (*M*s = 4.51 and 4.74; *SD*s = 1.62 and 1.27, respectively; *F*(1,51) = 0.323, *p* = .572, ƞ_p_^2^ = .006; 95% CI [-1.03, .58]). This last finding provides support for the validity of the solidarity with animals measure as not being tied to contacts with, or preferences for, a particular species of animals.

## Study 4b

Another group that may report particularly high levels of solidarity with animals are vegetarians. Indeed, while a diversity of motives exist for avoiding meat eating, including health concerns [87], some vegetarians make this lifestyle choice out of concern for the treatment of animals in the meat industry [88–89]. On this basis, we expected that vegetarians, on the whole, will report higher solidarity with animals compared to non-vegetarians. Given the variability of lifestyle choices with respect to meat-eating (e.g., some people will display their concerns for farm animals by minimising the quantity of meat they eat rather than becoming vegetarians per se), we also tested if lower frequency of meat-eating would be associated with greater solidarity with animals.

### Method

#### Measures

The same five items as in the prior studies were used to assess solidarity with animals (α = .95). Participants were also asked if they are vegetarian (yes/no). *Frequency of meat-eating* was assessed with one item: How many times per week do you eat meat (on average)?

#### Participants and procedures

The vegetarians recruited in Study 2 (*n* = 108) were compared to a random subsample of 108 non-vegetarian participants taken from that same study. The sample hence included 216 participants (177 women, *M*^age^ = 31.51, *SD* = 13.44). On average participants ate meat just under 3 times a week (*M* = 2.91, *SD* = 3.72). Participants provided their written informed consent (on a paper consent form) to participate in this study. The UQAM ethics committee approved this study.

### Results and Discussion

An ANOVA was conducted to compare vegetarians and non-vegetarians on the solidarity with animals measure. As expected, a significant difference was found between these groups, *F*(1,213) = 7.78, *p* = .006, ƞ_p_^2^ = .035; 95% CI [-.81, -.14], with vegetarians reporting higher solidarity with animals (*M* = 6.20, *SD* = 1.22) compared to non-vegetarians (*M* = 5.73, *SD* = 1.26). Also as expected, solidarity with animals correlated negatively with frequency of meat-eating (*r* = -.18, *p* = .009; 95% CI [-.32, -.05]). Attesting to their stability, these same findings were replicated when conducting the analyses on the entire sample of Study 2, with a correlation of *r* = -.15, *p* = .003; 95% CI [-.23, -.05] observed between solidarity with animals and frequency of meat-eating, and significant differences between vegetarians (*M* = 6.24, *SD* = 1.14) and non-vegetarians (*M* = 5.72, *SD* = 1.20) on the solidarity with animals measure (*F*(1,354) = 13.81, *p* < .001, ƞ_p_^2^ = .038).

## Human-Animal Similarity vs. Difference as Antecedent: Study 5

Study 5 aimed to test the causal associations between human-animal similarities and solidarity with animals. Given the central theoretical role played by intergroup similarities vs. differences, both in the intergroup relations literature [21] and in the context of human-animal relations [13], we specifically induced perceptions of similarity vs. dissimilarity between animals and humans. Indeed, increased phylogenetic similarities between animals and humans predicts more empathy toward animals [90], and more concern and distress when viewing animals mistreated by humans [32, 91]. We suggest that, consistent with identification with other human groups, viewing animals as more similar to humans should augment solidarity with animals.

We specifically relied on pictures of animals to induce similarity vs. dissimilarity of animals relative to humans given that the other possibility–i.e., making salient the similarity of humans relative to animals–could induce a sense of threat [47–48, 92]. A control condition, in which pictures did not pertain to either animals or humans (i.e., objects of urban architecture), was also included as a neutral baseline. Pilot testing confirmed that the animals in the similarity condition were indeed perceived as more similar and less different to humans compared to the animals in the difference condition, whereas all objects of the control condition were perceived as highly different from humans. Full details and findings for this pilot study are available from the authors upon request. For the main study, we expected that participants exposed to human-animal similarities would report higher solidarity with animals compared to participants exposed to human-animal differences and participants in the control condition.

### Method

#### Participants

Participants were 86 Canadian students from the Université du Québec à Montréal (46 women, *M*^age^ = 26.26, *SD* = 8.81). Participants provided their written informed consent (on a paper consent form) to participate in this study. The UQAM ethics committee approved this study.

#### Experimental manipulation

Participants were asked to complete a subjective processing of pictures task (e.g. [93](see also [94] for an example in the realm of human-animal relations). They were shown photos of animals or objects and were asked to write down what they saw in the picture, their general impression of the picture, and what title they would give the picture. Participants in the similarity condition were shown pictures of seven animals showing human-like facial expressions [95] (e.g., joy, contempt; www.timflach.com). In the difference condition, participants were shown seven pictures of the same species of animals but in contexts that emphasized their difference from humans (e.g., cow eating grass in a paddock). Participants in the control condition were shown seven pictures of architecture or urban objects (e.g., park bench). Presentation of the pictures was randomized in four orders. Order type did not have significant main (*F*(2,77) = 0.44, *p* = .728, ƞ_p_^2^ = .017) or interactive effects with condition (*F*(2,77) = 0.19, *p* = .904, ƞ_p_^2^ = .007). Three participants were removed due to reporting insight into the study’s aims.

#### Measures and manipulation checks

*Solidarity with animals* was measured with the five previously used items using a 1(*strongly disagree*) to 7(*strongly agree*) likert-type scale (α = .92).

Manipulation check measures were also included to assess: perceived status of animals relative to humans, legitimacy of this relative status, and explicit perceptions that animals are similar to humans. Perceived status was assessed with three items (α = .85): 1) “Are animals inferior to humans?” (measured on a scale from 1 = *animals are very much inferior to humans* to 7 = *animals are very much superior to humans*); 2) “Are humans superior to animals” (measured on a scale from 1 = *humans are very much inferior to animals* to 7 = *humans are very much superior to animals*–reversed score); 3) “Do animals have a higher or lower status compared to humans?” (measured on a scale from 1 = *humans are very inferior to animals* to 7 = *humans are very superior to animals*). Higher score indicated greater perceived status of humans compared to animals. Legitimacy of the relative status of humans and animals was measured with one item on a scale from 1(*not at all*) to 7(*completely*): “Think of the relative status that animals have relative to humans in our societies. Do you think this reflect the way things should be?”. Finally, participants provided their responses about the extent to which animals are similar to humans (on a scale from 1 = *not at all similar* to 7 = *completely similar*) and to which animals are different from humans (on a scale from 1 = *not at all different*) to 7 = *completely different*). The second items was reversed in order to compute the overall similarity score (α = .81). Higher score indicated greater perceived similarity between humans and animals.

A univariate ANOVA conducted on the explicit perceptions of human-animal similarity failed to reveal significant differences across conditions (*F*(2,83) = 1.014, *p* = .367, ƞ_p_^2^ = .024). This suggests that whereas the pictures were perceived as intended and elicited the expected effects on the solidarity with animals construct, participants’ perceptions of human-animal similarities appear to have been affected implicitly rather than explicitly by these pictures. The ANOVA conducted on the perceptions of status variable did however uncover significant differences across conditions (*F*(2,83) = 4.95, *p* = .009, ƞ_p_^2^ = .106). Helmert contrasts revealed that participants perceived a lower superior status for humans relative to animals in the similarity condition (*M* = 4.63, *SD* = 1.05) than in the other two conditions (difference condition: *M* = 4.86, *SD* = 1.03; control condition: *M* = 5.43, *SD* = 1.01; *p* = .026). Participants in the difference condition also reported lower superior status for humans relative to animals compared to participants in the control condition (*p* = .046). As well, in the ANOVA conducted on the perceptions that this relative status is legitimate (*F*(2,83) = 4.83, *p* = .010, ƞ_p_^2^ = .104), Helmert contrasts revealed that while participants in the similarity (*M* = 2.22, *SD* = 1.54) and in the difference condition (*M* = 2.25, *SD* = 1.42) did not differ significantly in terms of these perceptions (*p* = .107), participants in the control condition did report higher perceptions of status legitimacy (*M* = 3.40, *SD* = 1.94; *p* = .013) compared to participants in the difference condition.

### Results and Discussion

**Fig 1:**
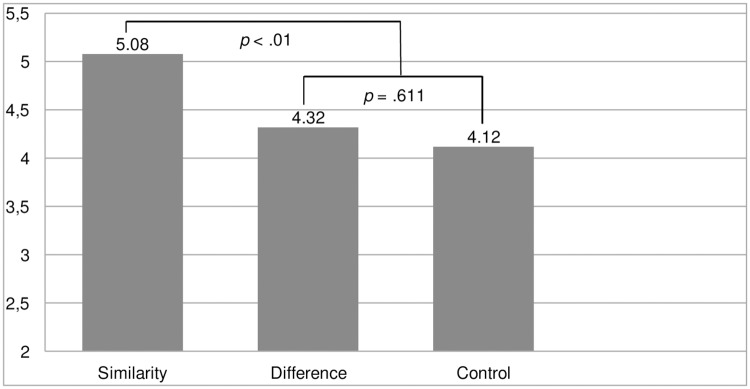
Means on Solidarity With Animals for the Conditions of Human-Animal Similarity, Human-Animal Difference, and Control (Study 5).

A univariate ANOVA revealed a significant overall difference across conditions on the solidarity with animals measure (*F*(2,83) = 3.95, *p* = .023, ƞ_p_^2^ = .087). Fig 1 presents the means for each condition. Helmert contrasts, which allow to compare the experimental group (e.g., the similarity condition) to the average of the other groups (i.e., the difference and the control conditions), were employed. This revealed that participants reported more solidarity with animals in the similarity condition (*M* = 5.08, *SD* = 1.39) than in the other two conditions (difference condition: *M* = 4.32, *SD* = 1.41; control condition: *M* = 4.12, *SD* = 1.43; *p* = .008; 95% CI [.25, 1.49]). Participants in the difference and control conditions did not differ significantly on the solidarity with animals measure (*p* = .611; 95% CI [-.75, 1.14]).

## Nomological Validity: Study 6

In Study 6 we assess the nomological validity of the solidarity with animals measure by simultaneously testing the associations between human-animal similarities as an antecedent of solidarity with animals and the consequences associated with solidarity with animals (e.g., lower speciesism). This integrative sequence of variables allows us to test, within the confines of the same mediating model, the identification process through which perceiving greater similarity between animals and humans leads to heightened concerns for animals. More specifically, this sequence assigns perceived similarity as a cognitive antecedent to the solidarity with animals construct (as was tested in Study 5 as well). Solidarity with animals in this sequence is conceptualized as a mediator that links these perceptions of similarity to more positive concerns and outcomes for animals. In this sense, solidarity with animals concretely represents an identification mechanism that connects us to other animals and which then facilitates taking and defending their perspective (i.e., as indexed by lower speciesism, lower justifications for the use of animals, and more moral concerns toward animals). This specific link between solidarity for animals and more positive animal-related outcomes was also tested in Studies 1 and will be tested further in Study 7.

### Method

#### Participants

Participants were 231 Canadian students at the Université du Québec à Montréal (177 female, *M*^age^ = 23.32, *SD* = 4.07). Participants provided their written informed consent (on a paper consent form) to participate in this study. The UQAM ethics committee approved this study.

#### Measures

*Human-animal similarity* was measured using two items: One item was positively worded (“To what extent are animals similar to humans?”; 1 = *Not at all similar*; 7 = *Completely similar*) and one item was reversed-scored (“To what extent are animals different from humans?”; 1 = *Not at all different*; 7 = *Completely different*). The scores were averaged with higher scores indicating more similarity of animals relative to humans (α = .79). *Solidarity with animals* (α = .94), *moral concerns toward animals* (α = .96), and *speciesism* (α = .88) were all assessed as in the prior studies. A new measure of animal-related outcomes assessed *justification for the use of animals* based on the uses of animals frequently and spontaneously reported [96]. Five positively worded items (e.g. “The use of animals for human purposes is necessary to ensure humans’ well-being”; “It is essential that humans eat animal meat to ensure our good health”) were measured on a 1(*do not agree at all*) to 7(*agree completely*) likert-type scale (α = .83).

### Results and Discussion

#### Correlations

We first inspected the correlations between the main variables. While perceiving human-animal similarity was related positively to solidarity with animals (*r* = .36, *p* < .001; 95% CI [.24, .48]), solidarity with animals was associated with more moral concerns toward animals (*r* = .53, *p* < .001; 95% CI [.42, .64]), lower justification for the use of animals (*r* = -.31, *p* < .001; 95% CI [-.43, -.18]), and lower speciesism (*r* = -.54, *p* < .001; 95% CI [-.62, -.41]). Perceived human-animal similarity also correlated with these outcomes following the same pattern (i.e., *r*s = .27; 95% CI [.14, .39], -.26; 95% CI [-.39, -.13], and -.36; 95% CI [-.45, -.22], *p*s < .001, for the moral concerns, justification, and speciesism variables, respectively).

#### Mediation analyses

We next conducted mediation analyses using multiple regressions and bootstrapping techniques to test the indirect effect of human-animal similarity on our three outcome variables (i.e., justification for the use of animals, moral concern toward animals, speciesism) via solidarity with animals. Focusing first on justification for the use of animals, regressions analyses revealed that human-animal similarity significantly predicted solidarity with animals (*β* = .36, *p* < .001), that solidarity with animals in turn was negatively associated with justification for the use of animals (*β* = -.25, *p* < .001; 95% CI [-.38, -.12]), and that the negative association between human-animal similarity and justification for the use of animals dropped from *β* = -.27, *p* < .001, to *β* = -.17, *p* = .010, after including solidarity with animals in the regression equation. The bootstrapped estimate of the mediation effect (using 5000 resamples, see [97]) confirmed that solidarity with animals was a significant mediator (IE = -.1039, CI 95% between -.1852 and -.0462).

We next focused on moral concern toward animals. The regression analyses revealed that solidarity with animals positively predicted moral concerns toward animals (*β* = .49, *p* < .001; 95% CI [.38, .62]), and that the positive association between human-animal similarity and moral concern toward animals dropped from *β* = .27, *p* < .001, to *β* = .09, *p*>.05, after including solidarity with animals in the regression equation. The bootstrapped estimate of the mediation effect (using 5000 resamples) confirmed that solidarity with animals was a significant mediator (IE = -1.2646, CI 95% between .7572 and 1.8021).

Finally, we focused on speciesism. Regression analyses revealed that solidarity with animals negatively predicted speciesism (*β* = -.47, *p* < .001; 95% CI [-.56, -.34]), and that the negative association between human-animal similarity and speciesism dropped from *β* = -.36, *p* < .001, to *β* = -.19, *p* = .001, after including the solidarity with animals construct in the regression equation. The bootstrapped estimate of the mediation effect (using 5000 resamples) confirmed that solidarity with animals was a significant mediator (IE = -.1382, CI 95% between -.2051 and -.0828). These mediation analyses confirmed the mediating role of solidarity with animals in the associations between human-animal similarity and lower prejudice towards animals.

## Predictive Validity: Study 7

In Study 7, we aimed to extend on the validation evidence provided in Studies 1 to 6 by directly examining whether solidarity with animals predicts bias in resource decision-making in line with expectations set out by social identity and self-categorization theories [21, 98]. According to this theoretical perspective, whether an individual is an ingroup or outgroup member has implications for how they are viewed relative to other ingroup members and the equity with which resources are allocated, including money, time, and even the protection of life. If people identify and feel solidarity with animals as part of their ingroup, then this should lead to reductions in bias toward humans compared to animals. To this end, we first examined whether solidarity with animals might predict basic indices of ingroup (humans) vs. outgroup (animal) bias using feeling thermometers and adjective ratings. Next we examined whether any reduction in bias might extend to decision-making in resource allocation dilemmas, where the needs of humans and animals are pitted against each other. We expected that people who feel solidarity with animals will rank the needs of animals more highly, even when these needs are directly opposed to the needs of fellow human ingroup members. As such, we focused on moral dilemmas, where the value of animal lives are pitted against the value of human lives, and a resource allocation dilemma, where limited funds are spread between charities that help humans (e.g., Oxfam, UNICEF) and animals (WWF, SPCA). In addition, we assessed participants’ willingness to engage in collective action on behalf of animals. Including these consequences allowed us to further test how solidarity with animals is consequential for both animals and humans.

We also tested these associations using a two-wave prospective design: Solidarity with animals was assessed at Time 1, and then, 5 months later at Time 2, the animal- and human-related consequences were assessed. Temporally separating these constructs allowed us to disentangle common method variance and provides a more stringent test of the associations between solidarity with animals and these outcomes. As such, we also included measures of speciesism, justification for the use of animals, and moral concern for animals at Time 2 as used in Studies 1 and 6.

### Method

#### Measures

*Solidarity with animals* was measured using the five items employed in the prior studies. Whereas solidarity with animals was assessed both at Time 1 (α = .91) and at Time 2 (α = .88; test-retest correlation of .79), the main analyses focused on Time 1 solidarity with animals predicting the Time 2 outcomes. This allowed us to reduce any common method variance and to observe the predictive validity of the solidarity construct over time. Participants also completed the previously-used measures of *moral concern toward animals* (α = .96), *justification for the use of animals* (α = .85), and *speciesism* (α = .91). To assess bias in favor of humans over animals and over pets, three *feeling thermometers* [99] were employed to assess participants’ feeling of warmth toward: animals, pets, and humans; two difference scores were created to capture bias in favor of humans relative to animals and to pets. Another measure of bias involved presenting a list including 17 positive *adjectives* (e.g., warm, intelligent, active) and asking participants to indicate how much each adjective applies to: humans (α = .93), animals (α = 90.), and pets (α = .92). An overall score that represents participants’ positive evaluation of each target was then created and two difference scores were computed to represent bias in favor of humans over animals and bias in favor of humans over pets.

A measure of *money donation* was also included. Participants were presented with the following information: ‘‘Assume you have a $100 budget that you decided to allocate to charitable organisations. Among the charities below, how would you distribute this $100?” Then, they were presented with a list of seven charitable organizations and their mandates (i.e., their goals and mission), and asked to indicate how much money they want to contribute to each: World Wildlife Fund for Nature (WWF); UNICEF; Amnesty International; Oxfam; Greenpeace; SPCA; Doctors without borders. They were asked to make sure the total amount they donated was $100. A variable was constructed that represented the difference in amount of donation made to charities focused specifically on human needs (i.e., UNICEF, Oxfam, Doctors without borders, Amnesty International) vs. those focused on animal needs (i.e., WWF, SPCA). Given that Greenpeace pertains specifically to nature (and somewhat indirectly to animals), donations to this organisation were left out of this calculation.

Three *moral dilemmas* [100] were presented to capture participants’ life and death decisions pitting the interests of animals (i.e., dogs) and humans against one another. Dogs were chosen as a specific animal given their commonality and closeness to humans [101] and the fact that they are not an endangered species, a factor which is known to come into play when making life and death decisions in moral dilemmas [100]. Participants’ responses in each dilemma were coded such that higher numbers indicate *more* bias in favor of saving human lives relative to saving animal lives. In the first dilemma, participants read about a trolley hurling down some tracks. They are an innocent bystander and need to decide to either: 1) Throw a switch, which will result in the death of the ten dogs on the side track (coded as 1); or 2) Do nothing, which will result in the death of the person (coded as -1). In the second dilemma, participants are provided with a similar trolley situation but the choices are either to: 1) Push the kennel over the bridge, which will result in the death of the ten dogs in the kennel (coded as 1); or 2) Do nothing, which will result in the death of the people in the trolley (coded as -1). In the third dilemma, participants are informed that a ship has sunk and that there are six individuals on a lifeboat: five humans and one dog. Because the lifeboat can only support five individuals, they need to decide between the following options: 1) Throw the dog over (coded as 1); 2) Draw lots among the humans and throw the losing human over (coded as -1); or 3) Draw equal lots and throw the loser among all six over (coded as 0).

To assess participants’ level of *animal-right activism*, the following item was used: ‘‘To what extent do you consider yourself to be an animal rights activist?” (1 = *not at all*; 7 = *extremely*). Five items (α = .85) assessed participants’ intentions to engage in *collective action* on behalf of animals in the next 6 months (i.e., Give money to a charity working for the interests of animals; Participate in demonstrations in favor of animal rights; Write to a politician to bring attention to animal-related issues (e.g., endangered species, treatment of animals in local pounds); Express yourself on the internet about animal-related issues; Sign a petition in support of animal rights; 1 = *very unlikely*; 7 = *very likely*).

#### Participants and procedures

The data for the Time 1 of this study was presented in Study 2. Five months after having completed the Time 1 questionnaire, participants were contacted by email to complete the Time 2 questionnaire. Participants were informed at Time 1 that they would be eligible for a draw of three cash prizes totaling $500CAD if they completed both questionnaires. A total of 162 participants completed both questionnaires (134 women, *M*^age^30.72, *SD* = 12.95), which implies an attrition rate of 56% from Time 1 to Time 2. Participants provided their informed consent at Time 1 by clicking on a box in the online consent form confirming that they had read the consent form and agreed to take part in the study. The UQAM ethics committee approved this study.

### Results and Discussion

Table 5 presents the descriptive statistics for the main variables and the correlations between solidarity with animals at Time 1 and the Time 2 consequences. Solidarity with animals at Time 1 correlated with all the consequences at Time 2 (i.e., 5 months later). Specifically, solidarity with animals was associated with lower speciesism and justification for the use of animals, but with more moral concern toward animals. Solidarity with animals was also associated with lower bias in favor of humans over animals and over pets on both the thermometer and on the adjective measures. In the three moral dilemmas, and over and above the general observed tendency to avoid actively harming another being, solidarity with animals was associated, in point-biserial (dilemmas 1 and 2) and bivariate (dilemma 3) correlations, with a lower tendency to favor humans over dogs. Solidarity with animals also correlated with higher levels of animal rights activism, greater intentions to engage in collective action on behalf of animals, and higher donations made to charities that seek to help animals relative to humans.

**Table 5:** Descriptive Statistics and Correlations between Solidarity with Animals at Time 1 and Human- and Animal-Related Outcomes at Time 2 (Study 7).

	Descriptive Statistics		T1 Solidarity with Animals
Variables	*M*	*SD*	
T2 Speciesism	5.23	1.16	.29***
T2 Moral Concern toward Animals	0.81	0.27	.32***
T2 Justifications for the Use of Animals	2.68	1.35	-.16*
T2 Adjectives in Favor of Humans over Animals	0.00	1.03	-.24**
T2 Adjectives in Favor of Humans over Pets	-0.23	1.08	-.23**
T2 Thermometers in Favor of Humans over Animals	-14.83	22.98	-.40***
T2 Thermometers in Favor of Humans over Pets	-19.46	25.03	-.35***
T2 Dilemma 1 Favoring a Human over Dogs	0.36	0.93	-.18*
T2 Dilemma 2 Favoring a Human over Dogs	-0.28	0.96	-.19*
T2 Dilemma 3 Favoring a Human over a Dog	0.00	0.82	-.17*
T2 Animal Rights Activism	3.86	1.70	.26**
T2 Collective Action Intentions on Behalf of Animals	4.06	1.72	.28***
T2 Donations to Human Charities over Animal Charities	2.65	45.33	-.27**

*M* = Mean; *SD* = Standard deviation.

* = *p* < .05.

** = *p* < .01.

*** = *p* < .001.

Across a 5-month period, the findings confirmed that solidarity with animals predicts a lower tendency to favor humans over animals, even on matters of life and death. Solidarity with animals also predicted a greater willingness to act on behalf of animals’ collective interests and a lower tendency to donate money to charities that seek to help humans relative to animals. Moreover, solidarity with animals was related to reduced prejudice against, and moral concern for, animals, consistent with Studies 1 and 6.

## General Discussion

While our interactions with animals are a common part of human life, the ways in which humans relate to and connect with animals have received less attention. In this research, we sought to contribute to the emerging field of human-animal relations [13] by investigating the nature of our psychological connection with other animals. To do so, principles from theories of intergroup relations [20–21] were applied to investigate a particular dimension of social identification, namely solidarity with animals. We also sought to understand the nature of solidarity with animals, the factors that trigger it, and to identify the consequences of this dimension of social identification on attitudes and behaviors that concern not only animals but also humans. To this end, we developed a measure of solidarity with animals and tested the measure’s validity, predictive utility, and antecedent factors across eight studies.

Attesting to its validity and utility, our findings indicated that the solidarity with animals measure presented a single-factor structure and that it was moderately to strongly endorsed, suggesting that on average, people do feel solidarity with other animals. The solidarity with animals measure also correlated with identification with nature and it predicted relevant animal-related outcomes (speciesism, moral concerns for animals) over and above other superordinate identities (Study 1). Solidarity with animals was found to be significantly associated yet conceptually distinct from dispositional empathy and openness to experience, as well as from measures of anthropomorphism and pet attachment (Study 2). Solidarity with animals also correlated negatively with different forms of prejudice (i.e., racism, ageism) and with hierarchy-enhancing ideological beliefs (SDO, RWA; Study 3). Also attesting to its construct validity, pet owners and vegetarians reported higher solidarity with animals compared to non-pets owners and to meat eaters (Studies 4a and 4b, respectively).

Providing causal evidence for the antecedents of the solidarity with animals construct and tapping into the underpinnings of this construct, Study 5 was experimental and investigated human-animal similarity (vs. difference) as causing greater (vs. lower) solidarity with animals. Further evidence for the role of human-animal similarities in predicting the solidarity with animals construct came from Study 6. This study also confirmed the applicability of an integrative mediation model, whereby solidarity with animals (as the identification mechanism that links us to other animals) is a mediator of the link between human-animal similarity (as a cognitive antecedent) and animal-related outcomes. Study 7 aimed to investigate how solidarity with animals predicts outcomes that are likely to impact on both animals and humans. This study also longitudinally replicated the links uncovered in the prior studies between solidarity with animals and the outcomes of speciesism, justification for using animals, and moral concerns toward animals. Importantly, Study 7 showed that solidarity with animals predicts lower bias in favor of humans in money donations and in life and death decisions when the interests of animals are in conflict with the interests of humans. These studies generally had large sample sizes and were hence well powered given the effect sizes observed.

Our research is grounded within the methods and fundamental postulates of intergroup theories [21], which can be applied to human-animal relations [10, 13, 32–33]. Herein, and given our interest in assessing the ways in which we may develop a sense of psychological connection to and ties with other animals, we specifically focused on the solidarity dimension of social identification [19]. Our work therefore extends our understanding of group processes and intergroup relations, by showing that these dynamics apply to our relationships with animals. In this sense, our findings confirm that if we consider the ‘‘other” as part of the self–be this other animal or human–their interests are going to be weighted more heavily in decision making. Our findings also have applied implications for understanding the use of animals as resources, as well as the management of scarce resources (including environmental resources) as they impact on the interests of both humans and animals. These are issues that will likely grow in importance over the coming decades as the human population continues to expand [102]. While the current work is tied to a more general movement–both scientific and social–toward understanding humans’ consideration for non-human species and other living entities (trees, plants), a full reconciliation of these different literatures goes beyond the scope of the current paper. Future work conducted in social psychology and in other fields should definitely keep these parallels in mind so as to build on, tie to these other literatures, and develop an integrated body of knowledge on the topic of human-animal relations.

### Limitations and Future Research Directions

Methodologically, responses to the solidarity with animals measure are self-reported, in line with a tradition in the social identity theory approach and with prior scales that assess social identification [19, 37–38, 40]. This assessment method also shows that people endorse items that explicitly link humans and animals in a direct and non-ambiguous manner. However, future research could employ other methods and techniques to further capture the solidarity with animals construct and extend these findings, such as by using implicit association techniques and reaction time measures that assess how much attributes assigned to the self overlap with attributes assigned to animals [103–104]. Future work could also employ behavioral (rather than self-reported) measures to more directly assess resource distribution between animals and humans (e.g., donating behavior [105]). While the current work relied on convenience samples, future confirmatory work could be conducted with representative samples also across a diversity of cultures (see [13]). Most of the studies presented herein also included a predominant proportion of female participants. Future work might seek to include more gender-balanced samples.

Conceptually, although our findings provide solid evidence for the consequences of solidarity with animals, as well as insight into the factors that predict this dimension of social identification, there is still much more to be learned about the construct. For example, little is known about the developmental origins of solidarity with animals. Another intriguing question concerns the impact of the broader intergroup context on our sense of solidarity with animals; apart from cognitive similarity, other and more macroscopic variables may also contribute to influencing solidarity with animals. For example, resources scarcity–in line with realistic conflict theory [106]–may impede a sense of connection with animals and promote a zero-sum struggle for resources. In contrast, framing intergroup relations as cooperative and mutually beneficial for both animals and humans may benefit human-animal relations and humans’ sense of belongingness with them. As well, past research has shown that reminders of death may lead people to deny their link to other animals [107]. Whether death threats may also reduce a tendency to feel connected to animals is an important question for future research.

Future research should also further test the interplay between two relevant identities that operate in the realm of human-animal relations, namely identification with humans and identification with animals. As mentioned in Study 1, the common ingroup identity model assumes relative independence of these two identities, with the superordinate (animal) identity leading to the most positive outcomes (as we have found throughout our studies). Other models of superordinate identification put forward alternative predictions, however. According to Hornsey and Hogg [108], to truly promote superordinate identification (i.e., with animals), one’s subgroup identity (humans) also needs to be considered and represented within the superordinate group (the animal kingdom). Future research is required to test these competing principles, to capture the conditions under which human and animal identities are compatible vs. incompatible with one another, and how the animal identity may represent a unique type of superordinate identity for humans.

Clearly, a wide array of important and exciting questions regarding the antecedents, nature, and consequences of solidarity with animals await investigation. It is hoped that our theorizing regarding the nature of the solidarity with animals construct, in conjunction with our new measure, will facilitate future efforts to answer these questions and will continue raising interest in a ubiquitous yet under-researched domain of human activity: our relations with non-human animals.
